# Retinal Thickness Associates with Cognition Dysfunction in Young Adult with Type 1 Diabetes in Taiwan

**DOI:** 10.1155/2022/9082177

**Published:** 2022-09-26

**Authors:** Jung-Lung Hsu, Pei-Shin Gu, Eugene Yu-Chuan Kang, Chi-Chun Lai, Fu-Sung Lo

**Affiliations:** ^1^Department of Neurology, New Taipei Municipal Tucheng Hospital, Chang Gung Memorial Hospital, Chang Gung University College of Medicine, New Taipei City, Taiwan; ^2^Department of Neurology, Chang Gung Memorial Hospital Linkou and Neuroscience Research Center, Chang Gung University College of Medicine, Taoyuan, Taiwan; ^3^Graduate Institute of Mind, Brain, & Consciousness, Taipei Medical University, Taipei, Taiwan; ^4^Brain & Consciousness Research Center, Shuang Ho Hospital, New Taipei City, Taiwan; ^5^Department of Pediatrics, Chang Gung Memorial Hospital Linkou, Chang Gung University College of Medicine, Taoyuan, Taiwan; ^6^Department of Ophthalmology, Chang Gung Memorial Hospital Linkou, Chang Gung University College of Medicine, Taoyuan, Taiwan; ^7^Graduate Institute of Clinical Medical Sciences, College of Medicine, Chang Gung University, Taoyuan, Taiwan; ^8^Department of Ophthalmology, Chang Gung Memorial Hospital, Keelung, Taiwan

## Abstract

**Background:**

Several factors could affect the cognitive dysfunction in patients with type 1 diabetes (T1D).

**Objectives:**

To report the characteristic of cognitive dysfunction in T1D and find its association with the retinal thickness.

**Subjects:**

We recruited one hundred and seven patients with T1D in our study.

**Methods:**

Detailed clinical and demographic factors and Cambridge Automated Neuropsychological Test Battery (CANTAB) were performed in all participants. The age at onset>5 years old and ≤5 years old groups was defined as old- and young-onset groups. The levels of the average values of 5-year glycated hemoglobin (HbA1c_5) before study were collected. Ophthalmic study and central retinal thickness (CRT) were performed.

**Results:**

The median age of T1D was 24.9 years old and 57 participants were women. The median age at onset was 7.4 years old, and mean disease duration was 17.2 years. After adjusting off multiple covariates by the regression analyses, the young-onset group had significantly a longer latency in sustained attention than old-onset group (*P* = 0.02). The HbA1c_5 showed a significantly negative association with the sustained attention (*P* = 0.03). The average values of CRT showed significantly negative correlations with the reaction time in sustained attention and visual searching (*P* = 0.04 and *P* < 0.01, respectively).

**Conclusions:**

Our results suggest that age at onset and glycemic control had significant impacts on different cognitive domains in T1D. The CRT had a significant correlation with sustained attention, which could be a surrogate markers of brain structural changes in T1D.

## 1. Introduction

Type 1 diabetes (T1D) can have a significant impact on the structure and function of developing brain [1, 2]. Several previous studies showed that there was a small to moderate degree of cognitive dysfunction in T1D patients compared with nondiabetic controls [3, 4]. Many cognitive domains had significant impairments in T1D compared with healthy controls, such as executive function [5], motor speed task [6], visuospatial ability, and memory function [7]. These cognitive impairments in T1D not only had increasing risk of academic underachievement but also had lower rates of school completion and work than healthy controls [8, 9]. Cognitive dysfunction could be associated with several factors, such as age at onset [10], disease duration [11], glycemic control [12], and microangiopathy [13].

Diabetic retinopathy is one of the important microangiopathic complications in T1D, and previous study showed that its prevalence was 15.5% [14, 15]. The associations between diabetic retinopathy and cognitive dysfunction had also been explored. One study that explored T1D with age 50 or more years showed that diabetic retinopathy was not associated with risk of dementia [16]. However, other studies showed that retinal vascular or neuronal changes were associated with cognitive dysfunction [17, 18]. Regarding the changes of brain structure, recently, Eriksson et al. had showed the associations between diabetic retinopathy and cerebral small vessel disease and microbleeds [19]. Besides, some studies had demonstrated the associations between the ophthalmic measurements and cognitive dysfunction in general population. The myopia [20], increased ocular axial length [21], and retinal thickness had been reported as significant factors related to cognitive dysfunction and brain volume decline [22]. Recently, retinal thickness had been reported to associate with cortex volume in multiple sclerosis [23]. Therefore, ophthalmic parameters may associate with the cognitive dysfunction in patients with T1D.

Traditional neuropsychological tests using paper and pencil methods had been applied in multiple medical disciplines including neurology, psychology, psychiatry, and primary care. On the other hand, computerized batteries can record reaction time aspects of performance that are difficult for psychometrists to achieve, and these may reflect activity in developing neural networks with more sensitivity that can be achieved with traditional tests [24, 25]. Cambridge Automated Neuropsychological Test Battery (CANTAB) is a well-designed and validated automatic neuropsychological test for pediatric neuropsychology [26]. In patients with T1D, the results of sustain, maintain, shifting attention and response time were significantly worse than controls [27]. However, there is no study showing the associations between the onset age, retinopathy, and cognitive dysfunction using CANTAB tests.

Recently, epidemiologic study in Taiwan had shown that from 2004 to 2015, T1D accounted for 0.51-0.59% of the entire diabetic population, the standardized incidence of T1D slightly decreased by 11%, and the standardized prevalence of T1D increased from 0.04% to 0.05% [28]. The verbal comprehension, perceptual reasoning, and working memory scores in the T1D group were significantly lower than in the controls [29]. However, the association between the age at onset, ophthalmic parameters, diabetic retinopathy, and cognitive dysfunction in T1D had not been explored. In the current study, we will explore the associations among the demographic factors, diabetic retinopathy, and cognitive dysfunction in our country. We also explore the associations between the ophthalmic variables in cognitive dysfunction. Our hypothesis is that age at onset and ophthalmologic parameters may relate to the cognitive dysfunction in patients with T1D.

## 2. Method

### 2.1. Subjects

Participants with T1D were enrolled in three tertiary centers in North Taiwan from Feb. 1994 to Aug. 2021. The study protocol was approved by the Institutional Review Board (CGMHIRB No. 201900821A3). Written informed consent was obtained from each participant before the study procedure. The diagnosis of T1D was based on the clinical diagnostic criteria [30]. In order to perform the neuropsychological tests, patients with significantly visual and auditory impairment or motor disability were excluded. Written informed consent was obtained from all participants before the study procedure. All methods were performed in accordance with the relevant guidelines and regulations. Detailed demographic data, clinical history, education years, body mass index (BMI), and biochemistry data were recorded. Disease duration was calculated from age at onset to study year. Laboratory studies included the following: creatine, high-sensitive C-reactive protein (HS_CRP), homocysteine, and average values of 5-year glycated hemoglobin (HbA1c) levels (HbA1c_5) and 10-year (HbA1c_10) levels before current study was collected. The sample size estimation was calculated based on the previous literature, and the estimated sample size was 94 with actual power of 0.95 [27]. To study the effect of age at onset on cognition, we further divided the patients with T1D into age at onset > 5 years old (old-onset group) and ≤5 years old groups (young-onset group) based on the previous literature [31, 32]. Besides, diabetic retinopathy was diagnosed based on the criteria described below, and we separated total participants as with and without diabetic retinopathy groups.

### 2.2. Cognitive Assessments

Cognitive assessment was performed by computerized neuropsychological tests based on Cambridge Automated Neuropsychological Test Battery (CANTAB) tests. CANTAB tests are language and culture free and then are suitable for application in different countries. Measurement of executive function over the life span was one of the advantages in the previous study using CANTAB tests [33]. In elementary school-age children, the internal consistency was acceptable, and 1-year stability was moderate to good for most tests [34]. CANTAB tests had been applied in several studies such as attention deficit/hyperactivity disorder [35], substance use [36], children with benign rolandic epilepsy [37], brain tumor [38], and T1DM [39]. In our study, eight cognitive domains were selected to study the cognition in T1DM participants: sensorimotor function and comprehension (MOT, motor screening task), processing and psychomotor speed (RTI, reaction time task), sustained attention (RVP, rapid visual information processing), visual searching (MTS, match to sample visual search), visual episodic memory (PAL, paired associates learning), working memory and strategy (SWM, spatial working memory), planning (OTS, one touch stockings of Cambridge), and emotion recognition (ERT, emotion recognition task). A well-trained researcher conducted the tests in a quiet room without the presence of parents. The testing procedure was explained to the participants, and then, the subjects sat behind a computer for a test run. Each assessment lasted about 1 hour, and depending on the subjects' perceived ability, a 5-minute rest was considered in the course of test execution.

### 2.3. Ophthalmic Measurements

Both eyes were given a full ophthalmic evaluation that included measurements of the best corrected visual acuity (BCVA) and was converted to logarithm of the minimum angle of resolution (logMAR) visual acuity (VA) for statistical analysis. Slit-lamp examination, indirect ophthalmoscopy, measurement of the spherical equivalent refractive error (KR-8100 Autorefractor; Topcon Corp., Tokyo, Japan), and value of axial length (AXL) (IOLMaster 700; Carl Zeiss, Jena, Germany) were also obtained. Two fields of 45-degree central field photography were taken using a fundus camera (Nonmyd *α*-DIII, KOWA, Tokyo, Japan) after pupil dilation. Optical coherence tomography (Spectralis, Heidelberg Engineering, Franklin, MA, USA) was performed, and the central retinal thickness (CRT) was measured to have the average retinal thickness within the central fovea circle of a 500 *μ*m radius. The values of CRT could be confounded by the macular edema, and in our study, no participant had macular edema. The severity of diabetic retinopathy was classified as grades 0 to 3 according to the Early Treatment of Diabetic Retinopathy Study [40–42]. The patients were followed up and treated following the guidelines of the American Diabetes Association (ADA) and the American Academy of Ophthalmology [43].

### 2.4. Statistical Analysis

All statistical analyses were performed using SPSS (version 21.0, Chicago, IL). Continuous variables were expressed as the median and interquartile range (IQR). Nonparametric tests by Mann-Whitney *U* tests and *χ*^2^ tests were performed in patients with different age at onset groups and with/without diabetic retinopathy groups whenever it is appropriate. Spearman's correlation analysis was performed to study the associations between clinical variables, ophthalmologic variables, and cognitive measurements. The values from CANTAB tests were performed log transformation to fit the normality tests for further analysis. Linear regression analyses were performed to adjust the age and disease duration in CANTAB tests in group comparisons. The potential presence of collinearity was assessed using variance inflation factor (VIF) < 4, and no collinearity was detected. Significant level was set as *P* < 0.05.

## 3. Results

A total of one hundred and seven patients with T1D were included in the current study. The mean age at study was 24.9 years old (range from 12.2 to 54.3 years old), and 57 (53%) participants were women. The median age at onset was 7.4 years old (range from 0.8 to 41.9 years old), and median disease duration was 17.2 years (range from 4.5 to 27.2 years).

### 3.1. Clinical and Ophthalmic Variables in Different Groups of Age at Onset

Table 1 shows the group comparison between age at onset > 5 years old and ≤5 years old groups. The median age, age at onset, education years, and disease duration had significant group differences (all *P* < 0.05). No significant group differences were found in BMI and laboratory studies. In ophthalmic parameters, no significant group differences were found in VA, logMAR VA, AXL, CRT, and severity of diabetic retinopathy.  Table 2 shows the medication usage in all T1D. All T1D patients used different regimen of insulin, and the median total daily dosage of insulin between different age at onset groups was not significant (*P* = 0.14). In CANTAB tests, no significant group differences were found by nonparametric tests. However, we used the multivariable linear regression to investigate the association of different T1D onset groups and the log-transformed CANTAB tests with adjustment of the age, disease duration, and education years. The mean response latency of RVP had significant group differences (*P* = 0.02). The young-onset group had significantly longer median response latency in sustained attention test than the old-onset group.

### 3.2. Clinical and Ophthalmic Variables between Patients with and without Diabetic Retinopathy

In diabetic retinopathy classification, we grouped the patient without diabetic retinopathy (severity = 0 in both eyes) and with diabetic retinopathy group (severity grade = 1 to 3 in either eye).  Table 3 shows the comparisons of clinical variables between the two groups. The median age, gender, age at onset, disease duration, and education years had no significant group differences. In laboratory studies, significantly higher values of HbA1c_5, HbA1c_10, and HS_CRP with lower values of serum creatine were found in patients with diabetic retinopathy group than without group (all *P* < 0.05). In ophthalmic study, no significant group differences were found in VA and CRT. Significantly lower values of logMAR VA and AXL in both eyes were found in patient with diabetic retinopathy group by nonparametric tests (all *P* < 0.05). In CANTAB tests, no significant group differences were found after log-transformed values of tests.

### 3.3. Associations between Clinical, Ophthalmic, and Cognition Variables

**Figure 1:**
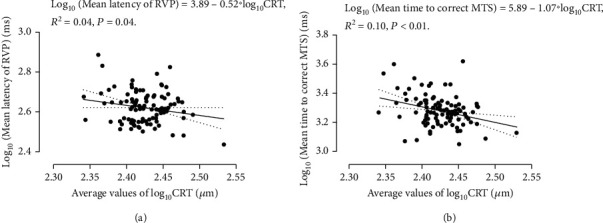
Regression analyses showed the significantly negative correlations between the average values of CRT and (a) mean latency of RVP test and (b) mean time to correct MTS test.

To study the associations between clinical parameters, ophthalmic parameters, and CANTAB tests, we firstly averaged the values of AXL and CRT from both eyes as ophthalmologic variables; then, we performed Spearman's correlation analyses.  Table 4 shows the correlation coefficient values from Spearman's correlation study. The HbA1c_5 showed a significantly negative association with the mean response latency of RVP (*P* = 0.03). The average values of CRT showed significantly negative associations with simple median reaction time of RTI, mean response latency of RVP, and median time to correct response of MTS (*P* = 0.03, *P* = 0.04, and *P* < 0.01, respectively). From Table 3, the HbA1c_5 had a significant group difference in patient with/without diabetic retinopathy. From regression analysis between the log-transformed HbA1c_5 and the mean response latency of RVP after stratified by the state of diabetic retinopathy, no significant correlation was found in patients with and without diabetic retinopathy (*P* = 0.07 and *P* = 0.38, respectively). In terms of the average values of CRT, the regression analysis was performed to study its correlation with the log-transformed simple median reaction time of RTI, mean response latency of RVP, and median time to correct response of MTS. The log-transformed average values of CRT showed significantly negative correlations with mean response latency of RVP and median time to correct response of MTS (*P* = 0.04 and *P* < 0.01, respectively) (Figure 1). In multivariate regression analysis, the log-transformed average values of CRT still showed significantly negative correlations with log-transformed mean response latency of RVP and median time to correct response of MTS after correcting off log-transformed age, disease duration, education years, and HbA1c_5 (*P* = 0.02 and *P* < 0.01, respectively).

## 4. Discussion

In the current study, we performed the automatic neuropsychological tests and ophthalmic measurements in a large number of young adults with T1D. Our findings suggest that age at onset showed a significant difference in mean response latency of RVP, which belongs to the attention domain of CANTAB tests. T1D with or without diabetic retinopathy did not show a significant group difference in CANTAB tests. Besides, the 5-year glycated hemoglobin (HbA1c) levels had a significantly negative association with sustained attention of CANTAB tests. Finally, the average values of CRT in patients with T1D had significantly negative correlations with mean response latency of RVP and median time to correct response of MTS, which are attributed to the sustained attention and visual searching domains of CANTAB tests. Our data using CANTAB test is compatible with the previous study which demonstrated that the attention domain and response domain had significant difference than controls [27]. Moreover, the association between the retinal thickness and cognitive dysfunction in young patients with T1D had not been reported before. Our results revealed that age at onset, disease duration, and retinal thickness in patient with T1D had significant associations with processing speed of attention and executive dysfunction.

Cognitive dysfunction in T1D has heterogenous results. From one systematic review, cognitive function changes in children/young adults had showed that some studies demonstrated impairment of executive function, motor speed tasks, and visuospatial ability, while others showed no significant differences [4]. On the other hand, the cognitive function changes in adults (usually ≥40 years old) had consistently reported decreased cognitive performance. From the previous literature, the early age at onset is an important factor for cognitive dysfunction in T1D [10]. In our study, we found that the age at onset had significantly affected the cognition demonstrated by the early age onset (age at onset ≤ 5years); patients had significantly decreased sustained attention domains. In the context of glycemic control, previous study showed the cognitive dysfunction as a consequence of high HbA1c [44]. In our study, all patients with T1D used different regimens of insulin. Different antidiabetic drugs may affect HbA1c in different ways, and different pleiotropic effects may alter the results because of improved outcome [45]. No significant difference of median total daily dosage of insulin was found between different ages at onset groups in our study. Higher HbA1c values had been reported to associate with moderate declines in motor speed and psychomotor efficiency [46]. In our study, although HbA1c had a significantly negative association with the mean response latency of RVP by Spearman's correlation study (*P* = 0.02), no significant correlation was found between the HbA1c values and the CANTAB tests after stratified by the state of diabetic retinopathy. These could be related to the relatively young age of our study participants (median age = 24.9 years old) and the small sample size. Future study will need to recruit more participants and age-matched controls to resolve the relationship between glycemic control and cognition in the young adult patients with T1DM.

A significant association between diabetic retinopathy and cognitive dysfunction was found in type 2 diabetes (pooled risk = 2.47) while did not identify such association between these two conditions in T1D [47]. In our study, we could not find a significantly cognitive dysfunction in patients with diabetic retinopathy group. In our study, shorter AXL and higher HbA1c levels in patients with diabetic retinopathy group had been reported before [48]. Previous study had shown that thinning of the retinal thickness had significant association with worse psychomotor speed and delay memory [17]. In our study, the average values of CRT had significant correlations with the attention domain of CANTAB tests even adjusted off multiple variables, which suggested that the more thinning of the retinal fiber, the worse response latency in attention test. Hilal et al. had demonstrated that in general population, the retinal vascular and neuronal parameters were associated with cortical microinfarct, and persons with both pathologies were likely to have cognitive impairment [49]. From UK biobank study, the retinal thickness had a significant association with total brain volume, gray matter, and white matter volumes [22]. In light of the above studies, our findings suggested that the CRT values link to attention and brain structure, which could be a surrogate marker of brain structure in patient with T1D. Its value could represent the response time of attention domains in neuropsychological tests. During the postpandemic era, telemedicine is a safe interactive system between patients and clinicians. Currently, technological improvements in telemedicine application have demonstrated efficacy and usefulness in screening the diabetic retinopathy [50]. Teleophthalmology is not only helpful in early detection but also in monitoring progression of retinal disease in diabetic retinopathy.

Several limitations should be addressed in the current study. First, our study excluded the patients with T1D who had significant visual and auditory impairment and motor disability, which may have a selection bias to more healthy participants in our study. We also did not recruit the age-matched controls in our study which could not demonstrate the difference of cognitive impairment between the patients with T1DM and controls. Nevertheless, we applied a relatively sensitive automatic neuropsychological test which showed the effect of age at onset and retinal thickness on cognitive dysfunction. Second, we did not perform the brain image study to explore the association between structure changes and cognitive dysfunction. Although we had explored the associations between risk factors of diabetic retinopathy in T1D in the previous study and found that old age at onset and higher HbA1c had significant correlations with diabetic retinopathy, we will further analyze the linkage between the brain structure, retinal thickness, and cognitive dysfunction in the future [51]. Third, several factors may contribute to the cognitive dysfunction such as HS_CRP, neuroinflammation markers, *β*-amyloid, and tau [52–54]. In our study, homocysteine and HS_CRP did not reveal associations with the cognitive dysfunction. Further study needs to include more inflammation markers in the future. Last, our study is a single-center nature of the study which limits the generalizability of our results.

## 5. Conclusion

We reported the results of CANTAB tests in young adult with T1D. Our findings suggest that early age at onset has significant contribution in impairment of attention domain than late age onset group. The retinal thickness in patients with T1D has a significant association with sustained attention domain, which could be a surrogate marker for brain structure changes. Based on our study, using computerized neuropsychological tests and measurement of central retinal thickness could be a feasible and reliable method to evaluate cognition in a large population of T1D.

## Figures and Tables

**Table 1:** Demographic, clinical, and ophthalmic variables between different groups of age at onset in patients with T1D in this study.

Variable	Age at onset ≤ 5 years old (*N* = 29), median (IQR)	Age at onset > 5 years old (*N* = 78), median (IQR)	*P* value
Age (year)	22.2 (19.7-25.5)	26.1 (23.5-29.3)	<0.01
Gender (M:F)	18 : 11	32 : 46	0.05
Age at onset (year)	2.3 (1.4-3.7)	9.4 (6.8-12.7)	<0.01
Disease duration (year)	20 (17-23)	15.9 (13.3-19.2)	<0.01
Education (year)	16 (12-16)	16 (12-16)	0.02
BMI (kg/m^2^)	22.5 (19.4-26.0)	22.5 (20.5-26.4)	0.66
HbA1c_5 (%)	8.1 (7.4-9.3)	7.8 (6.9-8.6)	0.07
HbA1c_10 (%)	8.5 (7.3-9.7)	8.2 (7.2-9)	0.15
Creatine (mg/dL)	0.63 (0.52-0.79)	0.66 (0.58-0.84)	0.27
HS_CRP (mg/L)	0.63 (0.26-1.67)	0.74 (0.36-1.94)	0.55
Homocysteine (*μ*mol/L	9 (7.0-10.4)	8.6 (7.1-10.5)	0.53
LogMAR VA_OD	0 (0-0.09)	0 (0-0.05)	0.28
LogMAR VA_OS	0 (0-0)	0 (0-0)	0.83
AXL_OD (mm)	24.7 (23.7-25.6)	24.7 (23.6-25.7)	0.85
AXL_OS (mm)	24.7 (23.9-25.5)	24.7 (23.6-25.6)	0.85
CRT_OD (*μ*m)	266.5 (256-278)	265 (255-282)	0.95
CRT_OS (*μ*m)	263 (256.5-282.5)	266 (255-278.7)	0.97
Severity of diabetic retinopathy_OD			0.83
0	21	63	
1	5	9	
2	2	4	
3	1	2	
Severity of diabetic retinopathy_OS			0.99
0	21	58	
1	5	13	
2	2	5	
3	1	2	

IQR: interquartile range; BMI: body mass index; HbA1c: glycated hemoglobin; HS_CRP: high-sensitive C-reactive protein; LogMAR VA: logarithm of the minimum angle of resolution visual acuity; AXL: axial length; CRT: central retinal thickness; OD: oculus dexter; OS: oculus sinister.

**Table 2:** Medication usage between different groups of age at onset in patients with T1D.

Variable	Age at onset ≤ 5 years old (*N* = 29), median (IQR)	Age at onset > 5 years old (*N* = 78), median (IQR)	*P* value
Insulin regimen			
1	23 (79.3%)	53 (68.0%)	
2	1 (3.4%)	12 (15.3%)	
3	1 (3.4%)	12 (15.3%)	
4	4 (13.8%)	1 (1.3%)	
Total daily dose (U/kg/day)	1.21 (0.98-1.36)	1.09 (0.89-1.31)	0.14

1: multiple-dose regimen with rapid and basal long-acting insulin. 2: premixed, short-acting insulin analogs given two to three times a day. 3: insulin pump. 4: regular and NPH given twice a day. IQR: interquartile range.

**Table 3:** Comparison of clinical and ophthalmic variables between patients with and without diabetic retinopathy in T1D.

Variable	Diabetic retinopathy group (*N* = 28), median (IQR)	No diabetic retinopathy group (*N* = 79), median (IQR)	*P* value
Age (year)	26.4 (23.3-29.4)	24.5 (21.7-27.7)	0.08
Gender (M:F)	6 : 12	39 : 40	0.22
Age at onset (year)	8.3 (4.4-11.8)	7.2 (4.2-11.6)	0.65
Disease duration (year)	17.8 (15.7-25.7)	16.7 (13.3-20)	0.12
Education year (year)	16 (16-16)	16 (12-16)	0.12
BMI (kg/m^2^)	24.8 (20.3-26.8)	22.4 (19.9-25.9)	0.38
HbA1c_5 (%)	8.7 (7.8-10)	7.6 (6.9-8.2)	<0.01
HbA1c_10 (%)	9.1 (8.2-10.4)	7.9 (7.1-8.7)	<0.01
Creatine (mg/dL)	0.63 (0.52-0.71)	0.69 (0.58-0.83)	0.03
HS_CRP (mg/L)	1.28 (0.42-2.3)	0.61 (0.27-1.54)	0.03
Homocysteine (*μ*mol/L)	8.4 (6.8-10)	8.8 (7.3-10.7)	0.23
LogMAR VA_OD	0 (0-0.09)	0 (0-0)	<0.01
LogMAR VA_OS	0 (0-0.15)	0 (0-0)	<0.01
AXL_OD (mm)	23.9 (22.9-25.1)	24.9 (23.9-25.8)	<0.01
AXL_OS (mm)	24.0 (22.9-24.9)	24.8 (23.9-25.8)	<0.01
CRT_OD (*μ*m)	260 (247-278)	267 (256-282)	0.12
CRT_OS (*μ*m)	263 (254-281)	266 (255-279)	0.70

BMI: body mass index; HbA1c: glycated hemoglobin; HS_CRP: high-sensitive C-reactive protein; LogMAR VA: logarithm of the minimum angle of resolution visual acuity; AXL: axial length; CRT: central retinal thickness; OD: oculus dexter; OS: oculus sinister.

**Table 4:** Spearman's correlation coefficients between CANTAB tests and clinical and ophthalmic variables.

Row	MOT (MOTML)	OTS (OTSPSFC)	PAL (PALTEA28)	RTI (RTISMDRT)	RVP (RVPML)	SWM (SWMBE4)	MTS (MTSRCAMD)	Disease duration	HbA1c_5	avgAXL	avgCRT
ERT (ERTRT)	0.15	-0.03	0.19^∗^	0.03	0.06	-0.01	0.32^∗∗^	0.16	0.14	-0.09	-0.15
MOT (MOTML)		-0.06	0.25^∗∗^	-0.03	-0.32^∗∗^	0.27^∗∗^	0.21^∗^	0.00	0.13	0.07	-0.15
OTS (OTSPSFC)			-0.14	-0.14	-0.08	-0.34^∗∗^	0.02	0.02	0.05	-0.02	0.00
PAL (PALTEA28)				0.02	-0.09	0.11	0.22^∗^	0.06	0.06	-0.04	-0.02
RTI (RTISMDRT)					0.33^∗∗^	-0.02	0.22^∗^	0.15	-0.00	0.00	-0.27^∗∗^
RVP (RVPML)						-0.07	0.09	0.06	-0.22^∗^	-0.06	-0.07
SWM (SWMBE4)							0.02	0.17	-0.15	-0.13	-0.16
MTS (MTSRCAMD)								0.17	-0.01	-0.12	-0.26^∗∗^
Disease duration									0.10	-0.20^∗^	-0.19
HbA1c_5										-0.15	-0.13
avgAXL											0.21

ERT: emotion recognition task; MOT: motor screening task; OTS: one touch stockings of Cambridge; PAL: paired associates learning; RTI: reaction time task; RVP: rapid visual information processing; SWM: spatial working memory; MTS: match to sample visual search; HbA1c_5: average values of glycated hemoglobin 5 years before the current study; avgAXL: average values of axial length; avgCRT: average values of central retinal thickness. Item in parenthesis represents the key parameter in its CANTAB test.

## Data Availability

The data that support the findings of this study are available on request from the corresponding author. The data are not publicly available due to privacy or ethical restrictions.
